# Risk factors and a novel cerebral infarction extent scoring system for postoperative cerebral ischemia in patients with ischemic Moyamoya disease

**DOI:** 10.1038/s41598-022-26985-3

**Published:** 2023-04-07

**Authors:** Yuanbing Chen, Xuan Gong, Zeng Yang, Fenghua Chen, Junyu Wang

**Affiliations:** 1 Department of Neurosurgery, Xiangya Hospital, Central South University, Changsha, Hunan China; 2 Department of Neurosurgery, The First People’s Hospital of Changde, Changde, Hunan China

**Keywords:** Neurology, Risk factors

## Abstract

Postoperative cerebral ischemic complication is the most common complication of revascularization surgery for patients with moyamoya disease (MMD). This retrospective study was conducted on 63 patients with ischemic MMD. Postoperative ischemia occurred in 15 of the 70 revascularization operations performed for patients after surgical revascularization, translating to an incidence of 21.4%. Univariate analysis revealed that onset infarction (*p* = 0.015), posterior cerebral artery involvement (*p* = 0.039), strict perioperative management (*p* = 0.001), interval time between transient ischemic attack (TIA) or infarction presentation and operation (*p* = 0.002) and preoperatively cerebral infarction extent score (CIES) (*p* = 0.002) were significantly associated with postoperative cerebral ischemia. Multivariate analysis revealed that strict perioperative management (OR = 0.163; *p* = 0.047), and preoperatively CIES (OR = 1.505; *p* = 0.006) were independently associated with postoperative cerebral ischemia-related complications. After comprehensive improvement of perioperative management protocol, the incidence of symptomatic infarction declined to 7.4% (4 out of 54). Analysis of the area under the receiver operating characteristic curve (AUROC) indicated CIES was a predictor for both postoperative ischemia and high follow-up modified Rankin Scale scores. In summary, strict perioperative management and CIES were identified as independent risk factors for postoperative ischemic complications in ischemic MMD, demonstrating that comprehensive and individualized perioperative management improve postoperative outcomes in patients with MMD. Furthermore, application of CIES to evaluate pre-existing cerebral infarction can improve the management of patients.

## Introduction

Moyamoya disease (MMD) is a rare cerebrovascular condition characterized with progressive steno-occlusion in the terminal segment of the internal carotid artery and its proximal branches and abnormally dilated compensatory collateral vasculature on angiography^1,2^. To date, there are no effective treatments for MMD but revascularization surgery has been shown to effectively prevent future ischemic strokes by improving cerebral blood flow, which improves patients’ life quality^3^. Postoperative complications, especially cerebral ischemic events after revascularization surgery, occur in 3.7–33.3% of the patients^4–9^. However, compared with standard revascularization procedures, the use of risk factors of postoperative ischemic complications and tailored perioperative management can result in better optimal surgical benefits to patients.

Studies have reported several risk factors for postoperative cerebral ischemic complications^5–7,10,11^. However, the specific risk factors of postoperative ischemic complications have not been determined. Furthermore, majority of previous studies included both ischemic and hemorrhagic MMD patients. However, there are significant differences in clinical presentations, mechanisms, and prognosis between ischemic and hemorrhagic MMD^12,13^. In addition, whether revascularization surgery can reduce recurrent intracranial hemorrhage or ischemic events in patients with hemorrhagic MMD remains controversial. In this study, we aimed to identify the risk factors for postoperative cerebral ischemic complications in only ischemic MMD patients. A novel cerebral infarction extent scoring system (CIES) for predicting postoperative cerebral ischemic complications was developed. In addition, a tailored perioperative management protocol, including both preoperative patient evaluation and postoperative management, for preventing immediate postoperative complications was developed.

## Methods

### Patient selection

All patients with ischemic MMD who received revascularization surgery in the department of neurosurgery of Xiangya hospital between June 2013 and August 2018 were enrolled in this study. These patients were from Hunan and Jiangxi provinces of the People’s Republic of China. The inclusion criteria were as follows: (1) a diagnosis of MMD was confirmed with six-vessel digital subtraction angiography (DSA) based on the criteria of the guidelines for diagnosis and treatment of MMD by the Research Committee on Spontaneous Occlusion of the Circle of Willis^14^; (2) initial attack as the infarction type, transient ischemic attack (TIA)-type, and frequent TIA-type were classified as ischemic MMD; and (3) patients who underwent indirect or combined revascularization surgery from June 2013 to August 2018. Patients with hemorrhagic MMD or moyamoya syndrome were excluded from this study.

All procedures conducted on human participants in this study were approved by the ethics committee of Xiangya Hospital of Central South University and conformed with the Declaration of Helsinki. All study participants gave written informed consent. The diagnosis of MMD was performed in accordance with the guidelines for diagnosis and treatment of MMD by the Research Committee on Spontaneous Occlusion of the Circle of Willis.

### Preoperative evaluation and surgical indication

Magnetic Resonance Imaging (MRI) and six-vessel DSA were performed within one week before surgery in all patients to confirm the presence of MMD. Specifically, MR imaging techniques (perfusion-weighted MRI (PWI) and diffusion-weighted imaging (DWI)) were used to assess regional cerebral perfusion and cerebral ischemic extent before surgery, respectively. Patients were indicated for revascularization surgery based on previous guidelines^14^. Briefly, symptomatic and hemodynamically affected hemisphere of the patients was first revascularized. Subsequently, contralateral hemisphere was revascularized at least three months after the first surgery. If the patient did not show cerebral ischemic symptoms associated with the contralateral hemisphere and contralateral cerebral perfusion decrease as revealed by PWI after the first surgery, subsequent surgery on the contralateral hemisphere was not performed.

### Cerebral infarction extent scoring system (CIES)

**Figure 1:**
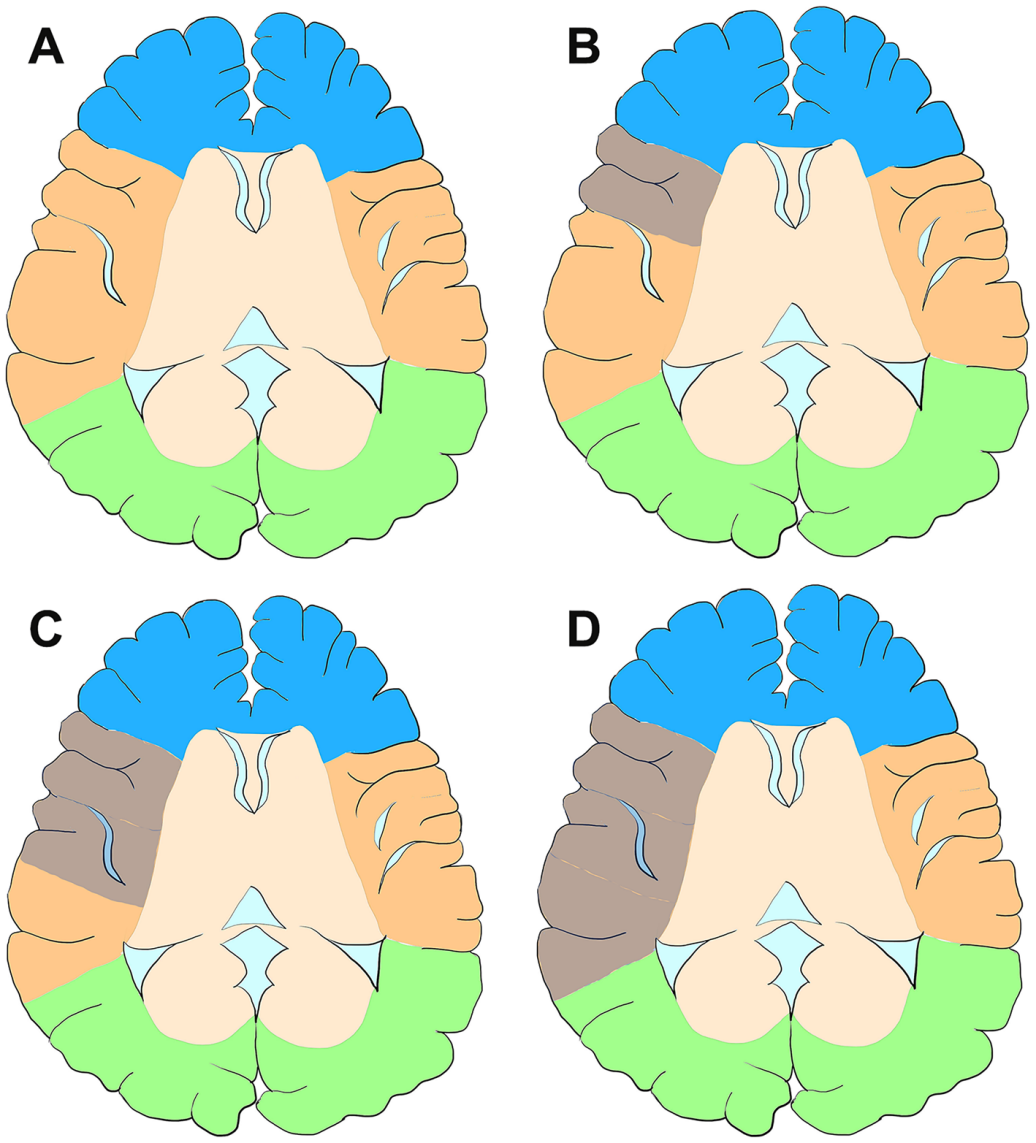
Schematic diagrams of CIES. (**A**) Diagrams showed no infarction preoperatively. CIES = 0 point, (**B**) diagrams indicated infarction (gray area) on the right temporal lobe, affecting about 1/3 of the lobe. CIES = 1. (**C**) Diagrams indicated infarction (gray area) on the right temporal lobe, affecting about 2/3 of the lobe, CIES = 2. (**D**) Diagrams indicated infarction (gray area) on the right temporal lobe, affecting about whole lobe. CIES = 3.

To evaluate the extent and severity of the preoperative cerebral infarction, we proposed a novel cerebral infarction extent scoring system. Based on the preoperative MRI features, including T1, T2 and DWI, we selected the slice with the largest area of infarction on MRI for evaluation. If multiple cerebral lobes were affected, the slice with the largest level of cerebral infarction in each lobe was selected. A score of 0 indicated no cerebral infarction while scores 1, 2, and 3 indicated an area of infarction less than one third of the cerebral lobe, between one third and two thirds of the lobe, and more than two thirds of the lobe, respectively. Finally, the score of each lobe was superimposed as the CIES (Fig. 1). In total, five lobes (frontal, temporal, parietal, occipital, and insula) in each hemisphere were assessed. Theoretically, the maximum score possible was 30 points.

### Surgery

In total, 63 patients with 70 revascularization operations were included in our study. All surgeries were performed by senior surgeons (Chen. F and Wang. J) following standardized procedures. Two surgical procedures were performed: indirect bypass (encephalon-dura-arterial-myo-synangiosis, EDAMS) and combined bypass (STA-MCA bypass with EDAMS). In brief, all surgical incisions were designed along the parietal branch of the superficial temporal artery (STA). Once the scalp was incised, the parietal branch of the STA was dissected to remove the surrounding fascia. The dura was opened in fan-shaped style, dura flaps were flipped and inserted under the edge of craniotomy to provide the possible collateral blood supply from a meningeal middle artery. If the recipient vessels matched the donor STA, a direct bypass was performed, which involved end-to-side anastomosis of the parietal branches of the STA to the cortical segment of middle carotid artery (MCA). Otherwise, indirect bypass was performed. Specifically, STA was sutured onto the surface of the hemisphere and multiple incisions were made on the arachnoid mater. Finally, the temporalis muscle flap was attached onto the surface of the brain and sutured to the edges of the folded dural flap. For STA-MCA bypass, intraoperative indocyanine green video angiography and postoperative magnetic resonance angiography (MRA) or computed tomography angiography (CTA) was routinely performed to confirm the graft patency.

### Perioperative management

**Figure 2:**
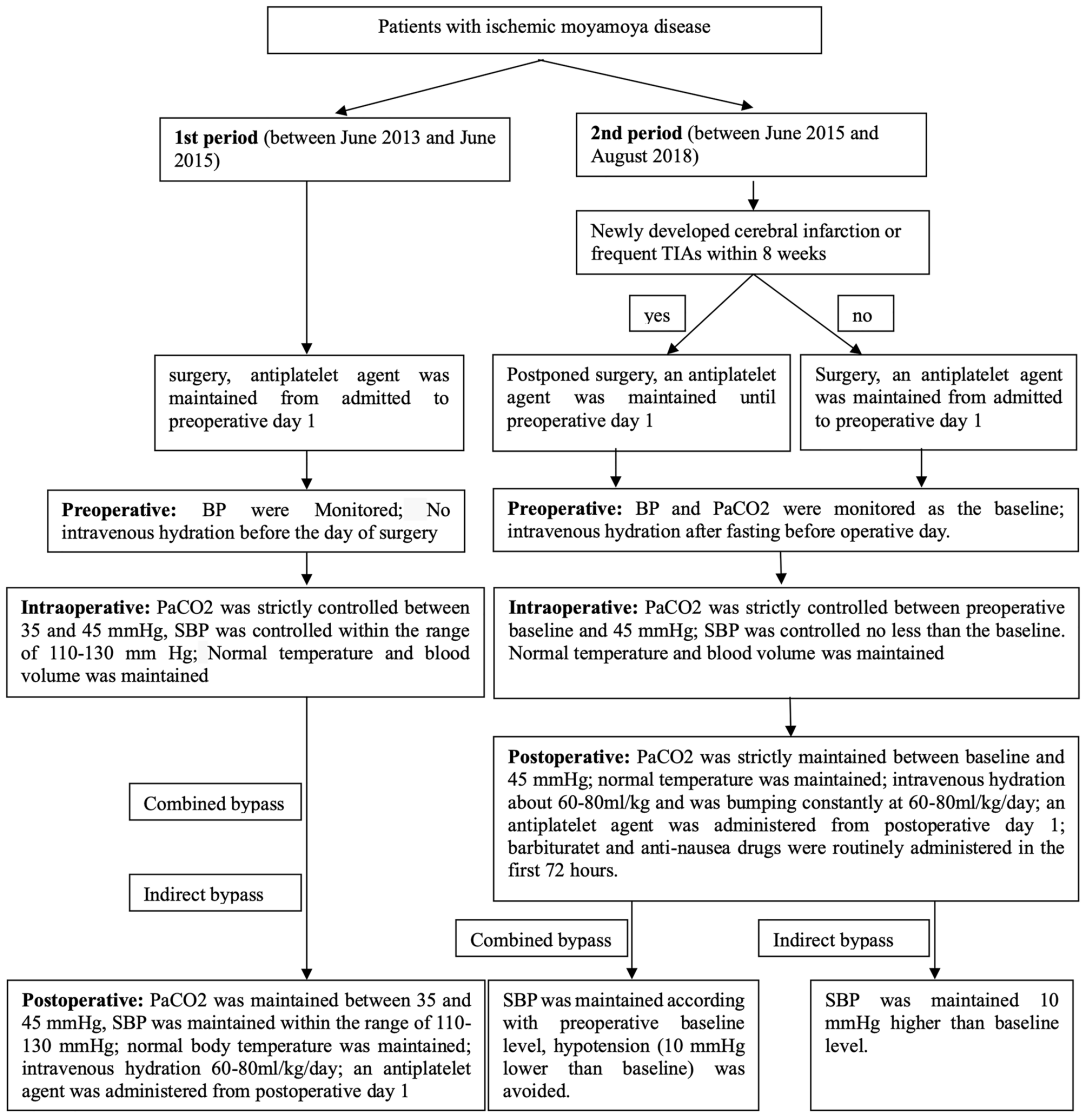
Protocol tree of perioperative management of patients with ischemic Moyamoya disease. BP: Blood pressure. *PaCO*_*2*_ partial pressure of carbon dioxide, *SBP* systolic blood pressure.

Considering the comprehensive improvement of perioperative management protocol after June 2015, perioperative management was divided into two unique periods. The first period was between June 2013 and June 2015. Perioperative management for this period involved surgery performed within 8 weeks (frequent TIAs were defined as those occurring more than three times per month) or not. In addition, the level of PaCO_2_ (partial pressure of carbon dioxide, arterial) and systolic blood pressure were strictly controlled within 35–45 mmHg and 110–130 mmHg, respectively. Normal body temperature was maintained throughout the perioperative period. In contrast, from June 2015 to August 2018, a strict perioperative management protocol was used. In this protocol, we delayed surgery for patients who had newly developed cerebral infarction (even asymptomatic) or frequent TIAs within 8 weeks. Preoperative intravenous hydration was continually administered from fasting before operative day to postoperative day 3. We routinely examined blood pressure and PaCO_2_ and maintained them based on the preoperatively level and the type of revascularization. The antiplatelet agent was discontinued on the day of surgery. The barbiturate and anti-nausea drug were routinely administered in the first 72 h. Details of this protocol are shown in the protocol tree (Fig. 2).

### Postoperative evaluation and clinical follow-up

**Figure 3:**
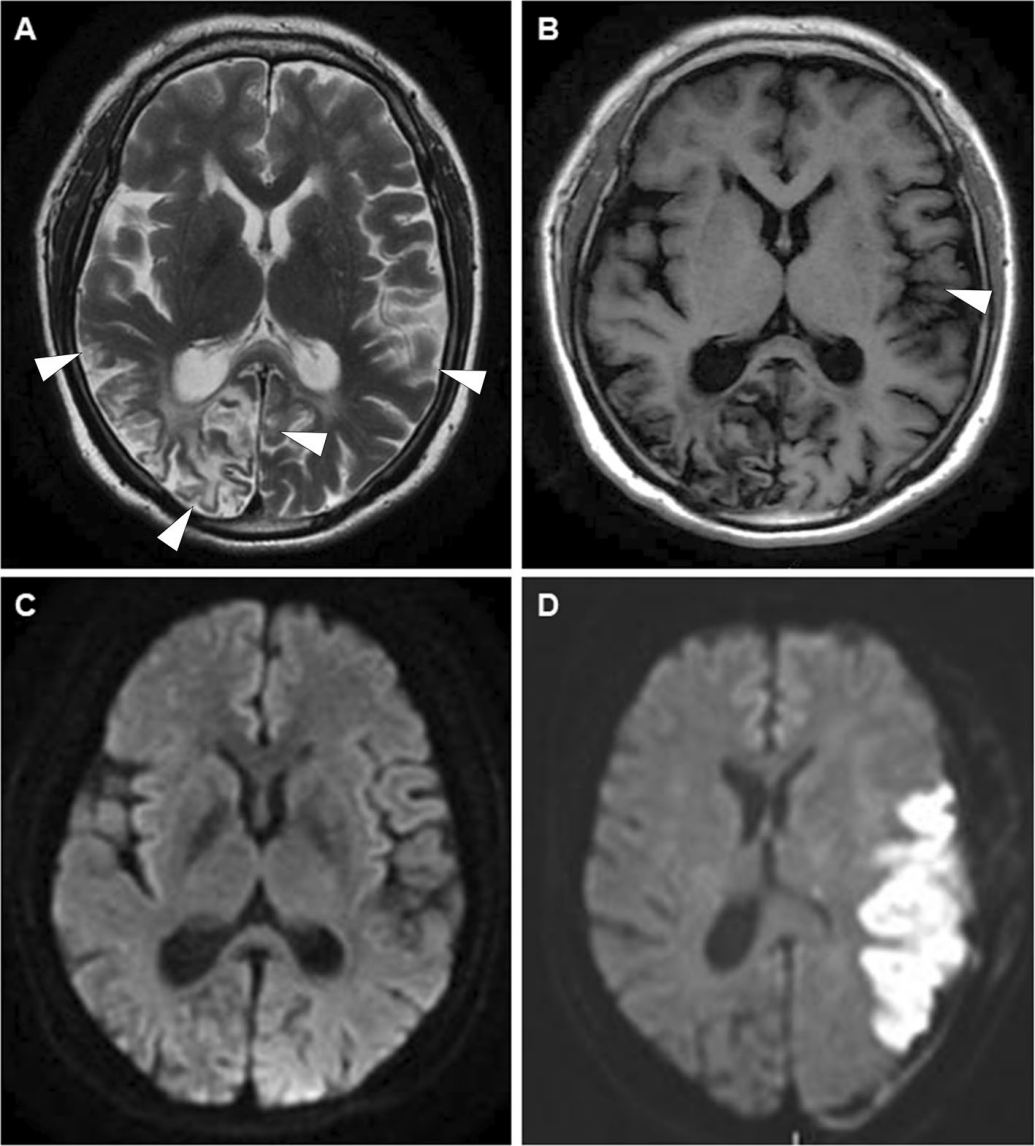
MR images obtained pre and postoperatively represent ischemic lesions. (**A**–**C**) T2-weighted, T1-weighted and diffused weighted MR images showed preoperative infarction in bilateral of temporal lobe and occipital lobe (white arrowheads). CIES = 7 point. (**D**) Diffused weighted MR images showed acute cerebral infarction in left temporal and occipital lobe postoperatively.

Postoperative CT scan was routinely performed to identify surgery-related hematoma or infarction on the operation day. Postoperative CTA and conventional MRI, including DWI were performed within one week after surgery. CTA was used to assess the patency of the bypass graft, whereas DWI was used to identify silent and symptomatic ischemic lesions. Repeat CT scan or DWI were performed in cases of new postoperative neurological deterioration (Fig. 3).

Patients were clinically evaluated at admission, at discharge and after three months follow-up. All outcomes were evaluated by a trained physician not directly involved in the care of these patients and blinded to the patient’s clinical data. Postoperative cerebral ischemic complication was defined as a symptomatic event of new cerebral infarction or asymptomatic infarction confirmed by CT or MRI within one week after surgery. Symptoms included focal neurologic deficit lasting > 24 h. Modified Rankin Scale scores (mRS) was used to evaluate functional outcomes and improvement.

### Statistical analysis

SPSS for Windows version 26.0 (IBM Corp) was used for statistical analysis. All categorical variables were presented as counts (with percentages) and analyzed with Pearson chi-square test. Two-tailed tests were used to compare continuous variables, which are presented as means ± standard deviations. Variables with a *p* < 0.05 on univariate analysis were included in multivariate analysis. The area under the receiver operating characteristic curve (AUROC) was calculated for the CIES as a predictor for postoperative ischemic complication as either good (0–2) or bad (3–6) mRS scores after three months follow-up. All values with *p* < 0.05 were considered statistically significant.

## Results

### Clinical characteristics and surgical outcomes

A total of 63 patients with ischemic MMD (26 females and 37 males) and mean age of 25.8 ± 20.1 (age range: 1–62 years) were consecutively recruited. Among them, 23 presented with TIAs and 47 cases underwent preoperative cerebral infarction. In addition, three patients had a history of hypertension and one had diabetes mellitus. Moreover, 32 and 22 patients were classified in stage 3 and stage 4, respectively, based on Suzuki stage. Notably, no patients with stage 1 or stage 6 was identified in our study. Totally 70 revascularization procedures, which included 50 indirect bypasses and 20 combined bypasses, were performed on 63 patients. In addition, imaging studies revealed bilateral MMD lesions in 54 out of 63 patients and unilateral MMD change in the rest. In 20 combined bypasses, all the STA-MCA anastomosis were successful as revealed by postoperative CTA or MRA. No patients died after surgical revascularization.

Postoperative complications occurred in 16 out of the 70 procedures (22.9%) performed, including 15 postoperative cerebral ischemic complications and one subdural hemorrhage. Postoperative infarction was confirmed with MRI within one week after surgery. Two patients showed ipsilateral infarction on MRI without any symptoms. Symptomatic ischemic complications occurred in 13 procedures (18.6%), including, ipsilateral infarction in nine patients and accompany with symptoms of various severity. Two patients had contralateral infarctions and another two had bilateral infarctions. The characteristics of postoperative ischemic complications are summarized in Table 1. After a three-month follow-up, the mRS in patients with postoperative cerebral ischemic complications was significantly worse compared with those without postoperative ischemic complications.

**Table 1:** Summary of 15 patients with postoperative cerebral ischemic complications.

No	Age/sex	Initial symptoms	Suzuki stages*	PCA involvement	Preoperative infarction or frequent TIA in 8 weeks	Operation	Strict perioperative management	Symptomatic/asymptomatic infarction	Onset of ischemic events after surgery	Postoperative cerebral infarction side
1	26/F	Infarction	IV	+	Yes	Indirect	No	Symptomatic	Within 24 h	Ipsilateral
2	40/M	Infarction	III	−	Yes	Combined	No	Symptomatic	Within 24 h	Contralateral
3	7/M	Infarction	III	+	Yes	Indirect	No	Symptomatic	Within 24 h	Bilateral
4	42/F	Infarction	II	+	Yes	Combined	No	Symptomatic	Within 24 h	Ipsilateral
5	2/M	Infarction	II	−	Yes	Indirect	No	Symptomatic	Within 48 h	Ipsilateral
6	6/M	Infarction	IV	−	No	Indirect	No	Symptomatic	Within 24 h	Ipsilateral
7	38/M	Infarction	II	−	No	Combined	No	Symptomatic	Within 24 h	Contralateral
8	44/F	Infarction	III	+	Yes	Indirect	No	Symptomatic	Within 48 h	Ipsilateral
9	5/F	Infarction	III	+	No	Indirect	No	Symptomatic	Within 24 h	Ipsilateral
10	50/F	Infarction	IV	+	No	Indirect	Yes	Symptomatic	Within 24 h	Bilateral
11	9/F	TIA	IV	−	No	Indirect	Yes	Asymptomatic	–	Ipsilateral
12	54/F	Infarction	V	+	No	Indirect	Yes	Symptomatic	Within 24 h	Ipsilateral
13	6/F	Infarction	III	+	No	Indirect	Yes	Symptomatic	Within 24 h	Ipsilateral
14	44/M	Infarction	IV	−	No	Indirect	Yes	Asymptomatic	–	Ipsilateral
15	28/M	Infarction	II	−	No	Combined	Yes	Symptomatic	Within 24 h	Ipsilateral

*Preoperative angiographic stages were evaluated using the criteria of Suzuki angiographic stage.

*PCA* posterior circulation artery, *TIA* transient ischemic attack.

### Risk factors of postoperative ischemic complication

**Figure 4:**
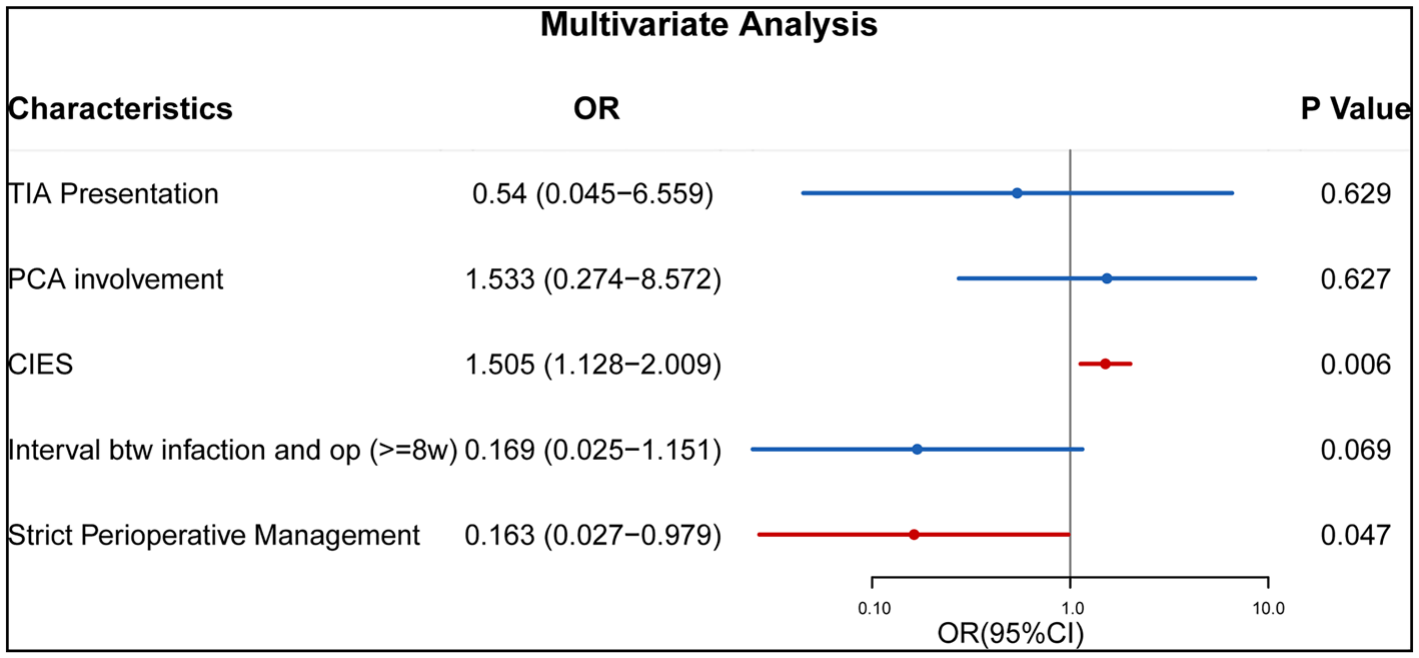
Multivariate analysis of postoperative ischemic complications. *CIES* cerebral infarction extent scoring system, *TIA* transient ischemic attack, *PCA* posterior cerebral artery.

To identify risk factors associated with the postoperative ischemia-related complications, we performed logistic regression analysis using clinical characteristics data. In the univariate analysis, initial symptoms (*p* = 0.015), interval between infarction and operation (*p* = 0.002), posterior cerebral artery involvement (*p* = 0.039), strict perioperative management (*p* = 0.001), CIES (*p* = 0.002) were associated with postoperative cerebral ischemia-related complications (Table 2). After adjustment for confounding factors in multivariate analysis, only strict perioperative management and CIES were found to be independent risk factors for postoperative cerebral ischemia-related complications in patients with ischemic MMD (Fig. 4). Five cases that underwent indirect bypass had postoperative cerebral ischemic complications during the strict perioperative management period compared with only one case in the combined bypass group. Although no significant differences observed by univariate analysis, these results indicated that patients who underwent indirect bypass were prone to postoperative infarction compared with those who received direct bypass. After strict perioperative management, the incidence of postoperative ischemic complication decreased from 52.9 to 11.3% with only four patients showing symptomatic infarction (7.4%). Furthermore, after the strict perioperative management, 15.1% (8/53) of the cases exhibited symptom improvement based on discharge mRS compared with 5.9% (1/17) in first period.

**Table 2:** Clinical characteristics of MMD patients.

Characteristic	All Pts (n = 70)	Post-op ischemic complications		*p* value
		Absent (n = 55)	Present (n = 15)	
**Mean age at op (years)**	25.8 ± 20.1	26.0 ± 20.6	25.3 ± 19.0	0.904
Children	36	30	6	0.386
Adult	34	25	9	
**Gender**				
Male	44	37	7	0.143
Female	26	18	8	
**Onset symptoms**				
TIA	23	22	1	0.015
Infarction	47	33	14	
PCA Involvement	22	14	8	0.039
**Suzuki stage**				
1	0	0	0	0.662
2	13	9	4	
3	32	27	5	
4	22	17	5	
5	3	2	1	
6	0	0	0	
CIES	3.3 ± 3.3	2.4 ± 2.4	6.5 ± 4.0	0.002
**Interval btw infarction and op**				
< 8w	20	11	9	0.002
> = 8w	50	44	6	
**Type of surgery**				
Indirect bypass	50	39	11	0.854
Combined bypass	20	16	4	
Operating duration	166 ± 97.9	158.4 ± 88.3	194.0 ± 126.9	0.321
Strict Peri-op management	53	47	6	0.001
mRS at 3 mths Post-op	1.9 ± 1.3	1.5 ± 0.9	3.4 ± 1.5	< 0.001

*CIES* cerebral infarction extent scoring system, *Interval btw infarction and op* Interval time between frequent TIA or infarction presentation and operation, *mRS* modified Rankin Scale, *TIA* transient ischemic attack, *PCA* posterior cerebral artery, *Peri-op* perioperative, *Post-op* postoperative.

Even though no significant difference was observed between pediatric and adult patients in postoperative ischemic complications. We performed the univariate analysis in adult and pediatric patients separately. We found the strict perioperative management and CIES were associated with the postoperative cerebral ischemic complication both in pediatric and adult group (supplementary Table S1 and Table 2).

### Assessment of the CIES system

**Figure 5:**
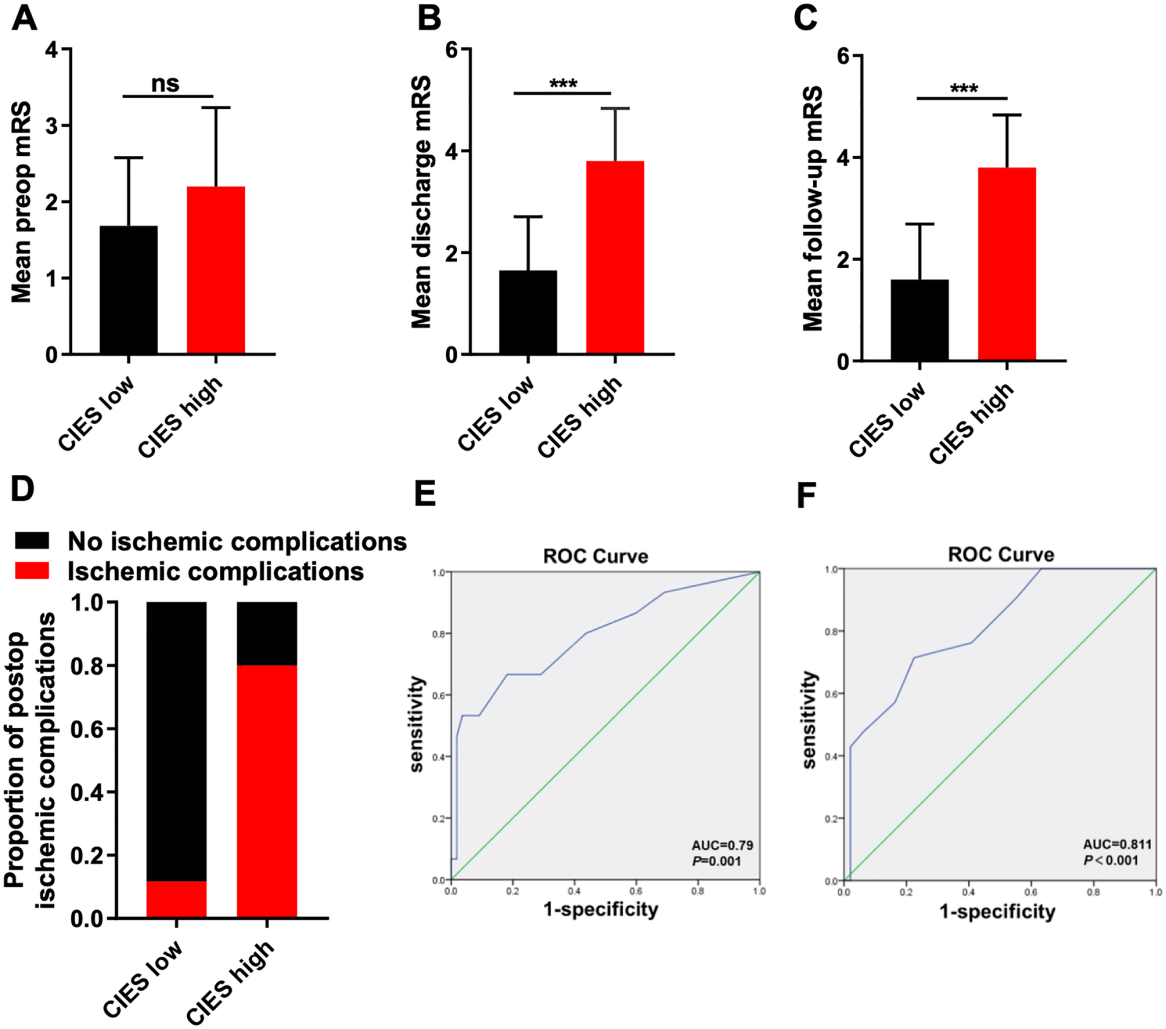
Preoperative, postoperative and follow-up mRS based on the high or low CIES. (**A**) Comparison of preoperative mRS between low (< 7) and high (≥ 7) CIES subgroups (ns: not significant, Mann–Whitney U test). (**B**) Comparison of discharge mRS between low and high CIES subgroups (***p < 0.001, Mann–Whitney U test). (**C**) Comparison of follow-up mRS between low and high CIES subgroups (***p < 0.001, Mann–Whitney U test. (**D**) The difference of incidence of postoperative ischemic complications between low and high CIES subgroups. (**E**,**F**) AUROC for CIES as a predictor for postoperative ischemia (**E**) or a high follow-up mRS score (**F**).

We then evaluated the association between CIES and mRS. The patients were divided into low CIES (< 7) and high CIES (≥ 7) groups. Results showed that both the mean discharge mRS and mean follow-up mRS were significantly higher in the high CIES group than in the low CIES group, but no difference was found in preoperative mRS. ROC curve was calculated to validate the predicted value of CIES in this cohort. AUROC of 0.790 and 0.811 were found for post-operative cerebral ischemia complications and high follow-up mRS, respectively (Fig. 5).

## Discussion

Moyamoya disease (MMD) is an idiopathic disease, which disproportionately affects East Asian populations^15^. A key feature of MMD is its bimodal distribution with age, with the largest peak being among patients aged 35–45 years, followed by those aged 5–9 years^16^. Revascularization surgery has been shown to effectively prevent future ischemic events and improve the quality of life of pediatric and adult patients^17,18^. Although similar efficacy has been reported in pediatric patients, evidence for the differences in outcome between direct bypass and indirect bypass in adult patients with MMD is inconclusive. Previous studies have reported that direct procedure is more effective than indirect bypass in adult patients, although the difference between the two approaches is not significant in terms of perioperative ischemia^14^. Another study demonstrated that direct bypass was more effective in preventing recurrent ischemic stroke than indirect bypass for adult ischemic MMD^19^. Other studies have found opposite results. One study revealed that recurrent ischemic-stroke occured more frequently in direct bypass than in indirect bypass^20^. Elsewhere, it was suggested that direct bypass and indirect bypass may have similar outcomes in adults MMD patients but indirect bypass was preferred because of its simplicity and safety. Interestingly, the postoperative infarction rate in their study was much higher in indirect bypass (14.7%) group than that in direct bypass (4.7%)^21^. A large study which enrolled 358 consecutive procedures in a single university found that there were no differences in the postoperative stroke rates between the direct and indirect procedures. However, after reviewing literatures, they concluded that revascularization was significantly more frequent in direct bypass than in indirect bypass based on postoperative angiography^22^. In our view, patients who undergo combined bypass could benefit from both immediate flow restoration from STA-MCA anastomosis (direct bypass) and pial synaiosis in the long run (indirect bypass). Therefore, in our study, we considered combined surgery as the first choice for all adults patients, which is consistent with other studies^23,24^. For all 34 adult patients, 20 underwent direct bypass surgery, whereas the other 14 patients underwent indirect bypass surgery. Among them, six patients had no appropriate recipient vessels to perform the direct bypass in the sulci, and thus we did not dissect the sylvian fissure to find adequate arteries. Moreover, direct bypass was not achieved in two patients because of repeat intraoperative thrombi formation at the anastomosis site during the procedure. Consequently, we converted to indirect bypass after several attempts to avoid severe postoperative ischemia-related complications. Furthermore, another six patients declined combined surgery because of safety concerns. As for pediatric patients, we preferred indirect bypass.

Cerebral ischemia is a common postoperative complication in patients with MMD and maybe the major factor inducing postoperative neurological deterioration^24^. Previous studies have reported that the incidence of postoperative cerebral ischemic complication after revascularization surgery varies from 3.7 to 33.3%^7,10,22,25^. The mechanism underlying the development of postoperative ischemic complications is complex. Postoperative SPECT indicated dynamic change in cerebral hemodynamics after direct bypass, suggesting that the so-called watershed shift may contribute to postoperative cerebral ischemia^26^. Other possible factors that may induce ischemia after surgery include sacrificing small penetrating branches of the recipient artery, vasospasm, acute occlusive changes in major cerebral arteries, and a decrease in regional cerebral blood flow and cerebral perfusion caused by hypotension during anesthesia^22,27,28^.

Evaluation of the risk factor and performing an optimal revascularization procedure under a tailored perioperative management will ameliorate prognosis and benefit the patient^29^. Higher Suzuki stage and preoperative ischemic presentation have been suggested as independent risk factors for newly ischemic events after revascularization surgery^10,30^. Other factors such as younger age (≤ 3 years), presence of underlying disease, female sex, TIAs frequency, and recent stroke episode have also been reported to be associated with postoperative ischemic complication^30–32^. Our pilot study indicated that preoperative infarction may be associated with postoperative ischemic events, which is in agreement with previous studies^10,31^. However, compared with patients who presented TIA preoperatively, we found that patients with mild preoperative infarction (infarction extent less than 2 brain lobes) showed no between-group differences in postoperative ischemic complications (data not shown). To better describe the severity of cerebral infarction, we proposed a CIES grading system and established that CIES is an independent risk factor for postoperative ischemic complications. This could be explained by the higher vulnerability of the delicate hemisphere to hemodynamic instability induced by revascularization surgery, increasing the risk of infarction. We also validated that high CIES (≥ 7) as an effective predictor of high rate of postoperative ischemic complications and high discharge mRS. On the contrary, patients with low CIES (< 7) tended to have a more favorable postoperative outcome. Recently, a new MMD grading system was reported by a Berlin group. They predicted clinical symptoms based on DSA findings and features of chronic cerebrovascular insufficiency (MRI and Xenon-CT). Although the Berlin grading system was valuable for stratifying preoperative symptoms of adult patients, there were no statistically significant differences when correlating with clinical postoperative stroke^33,34^.

Patients with larger cerebral infarction usually have worse mRS. In our study, patients with higher CIES showed higher discharge mRS and worse outcome at three-month follow-up. However, there was no statistical difference in preoperative mRS between low and high CIES groups, which may be due to the strategy of delaying revascularization surgery for eight weeks after an acute stroke or asymptomatic infarction revealed by MRI. During this period, the reconstruction of cerebrovascular reserve capacity and the recovery of neurological function resulted in improvement mRS, although the brain infarction persisted^35^.

Several researchers have demonstrated the efficacy of perioperative management protocols^5,29^. However, none of these protocols can provide comprehensive perioperative management for all ischemic MMD patients. Rashad et al. used their protocol only with patients who experienced direct bypass. Muraoka et al. described perioperative management merely for pediatric patients. Previous studies found that cerebral infarction and frequent TIAs may be indicators of the instability of cerebral hemodynamics condition in both adult and pediatric patients^4,6,31^. As mentioned above, we recommend postponement of revascularization surgery for patients with a recent cerebral infarction for eight weeks, since the period to reconstruct hemodynamics stability is at least 6–8 weeks^6,7^. Antiplatelet treatment was report to prevent emboli formation, thus improving cerebral perfusion, and stabilize the hemodynamics to reduce the risk of ischemic stroke and transient ischemic attack^36,37^. Thus, antiplatelet was routinely administered and only suspended on the day of operation in our series. Another report suggested that the antiplatelet agent was discontinued 7 days prior to the surgery and re-administered 3 days after surgery^22^. Neither previous studies nor our study found adverse effects of aspirin on intracranial hemorrhage during perioperative period^5,22^.

Symptomatic postoperative cerebral hyperperfusion syndrome is also a common complication in patients with direct bypass surgery due to regional cerebral blood flow increasing through STA-MCA direct anastomosis^6,10^. Unlike the strict blood pressure lowering strategy in the first period, our postoperative blood pressure management is more flexible during the second period. In the patients with combined bypass, postoperative systolic blood pressure was maintained at 10 mmHg less than the preoperative baseline to avoid hyperperfusion syndrome; whereas in patients who underwent indirect bypass, cerebral blood flow did not improve immediately after surgery. Therefore, blood pressure was maintained at about 10 mmHg higher than the preoperative baseline blood pressure to antagonize cerebral vasospasm and maintain the normal cerebral perfusion. Where there is an increased risk of cerebral ischemia symptoms associated with hypocapnia induced by hyperventilation, strict control of perioperative PaCO_2_ is uncontroverted, but the range of management standards remains unclear^38–40^. In our protocol, PaCO_2_ was monitored in every patient and strictly controlled between each preoperative baseline level and 45 mmHg both intraoperatively and postoperatively. The barbiturate was used as a sedative drug for patients to prevent hyperventilation with crying and exercise, especially in children. Barbiturate was also reported useful for controlling increased intracranial pressure, reducing cerebral damage of focal cerebral ischemia and minimize cerebral edema^5,41–43^. The swelling of temporalis muscle on the lateral brain surface and the change of blood flow increases the risk of increasing the seizure after surgery. Nausea is the most common postoperative complication associated with the anesthetic agents, and can easily cause hyperventilation. Thus, anti-epilepsy and anti-nausea drugs were routinely administered in the first 72 h. After adopting strict perioperative management in second period decreased the incidence of postoperative cerebral ischemia to 11.3%, and when only symptomatic infarctions are considered, the rate was only 7.5%. which is lower than most of previous studies^6,35,44^.

### Limitation

This study has some limitations. Firstly, this was not a randomized study, which could have led to selection bias. Secondly, although we included numerous clinical factors, several other preoperative characteristics, including comorbidities and preoperative hemodynamic state, were not analyzed in this study. Additionally, it would be valuable to analyze risk factors of postoperative complications in pediatric and adult patients separately in the future.

## Conclusion

This study demonstrates that postoperative ischemic complications in ischemic MMD patients can be significantly decreased to a favorable level after strict perioperative management. Furthermore, a CIES scoring system to precisely evaluate the pre-existed cerebral infarction was developed for predicting postoperative ischemia-related complications in MMD patients.

## Supplementary Information

Supplementary Information.

## Data Availability

Restrictions apply to the availability of data generated or analyzed during this study to preserve the confidentiality of the participants, but are available from the corresponding author on reasonable request.

## References

[1] Scott RM, Smith ER (2009). Moyamoya disease and moyamoya syndrome. N. Engl. J. Med..

[2] Ihara M (2022). Moyamoya disease: Diagnosis and interventions. Lancet Neurol..

[3] Acker G, Fekonja L, Vajkoczy P (2018). Surgical management of Moyamoya disease. Stroke J. Cereb. Circ..

[4] Funaki T (2015). Unstable Moyamoya disease: Clinical features and impact on perioperative ischemic complications. J. Neurosurg..

[5] Muraoka S (2018). Postoperative cerebral infarction risk factors and postoperative management of pediatric patients with Moyamoya disease. World Neurosurg..

[6] Park W, Ahn JS, Lee HS, Park JC, Kwun BD (2016). Risk factors for newly developed cerebral infarction after surgical revascularization for adults with Moyamoya disease. World Neurosurg..

[7] Kim SH, Choi JU, Yang KH, Kim TG, Kim DS (2005). Risk factors for postoperative ischemic complications in patients with moyamoya disease. J. Neurosurg..

[8] Yu L (2019). Revascularization surgery in patients with ischemic-type Moyamoya disease: Predictors for postoperative stroke and long-term outcomes. World Neurosurg..

[9] Choi JW (2020). Postoperative symptomatic cerebral infarction in pediatric Moyamoya disease: Risk factors and clinical outcome. World Neurosurg..

[10] Zhao, M. *et al.* Risk factors for and outcomes of postoperative complications in adult patients with Moyamoya disease. *J. Neurosurg*. 1–12. 10.3171/2017.10.JNS171749 (2018). 10.3171/2017.10.JNS171749 29600916

[11] Deng X (2021). Risk factors for postoperative ischemic complications in pediatric moyamoya disease. BMC Neurol..

[12] Okada Y (1998). Effectiveness of superficial temporal artery-middle cerebral artery anastomosis in adult moyamoya disease: Cerebral hemodynamics and clinical course in ischemic and hemorrhagic varieties. Stroke.

[13] Houkin, K., Kamiyama, H., Abe, H., Takahashi, A. & Kuroda, S. Surgical therapy for adult moyamoya disease. Can surgical revascularization prevent the recurrence of intracerebral hemorrhage? *Stroke* **27**, 1342–1346. 10.1161/01.str.27.8.1342 (1996). 10.1161/01.str.27.8.1342 8711799

[14] Research Committee on the, P., Treatment of Spontaneous Occlusion of the Circle of, W. & Health Labour Sciences Research Grant for Research on Measures for Infractable, D. Guidelines for diagnosis and treatment of moyamoya disease (spontaneous occlusion of the circle of Willis). *Neurol. Med. Chir. (Tokyo)* **52**, 245–266. 10.2176/nmc.52.245 (2012). 10.2176/nmc.52.245 22870528

[15] Ahn IM (2014). Incidence, prevalence, and survival of moyamoya disease in Korea: A nationwide, population-based study. Stroke.

[16] Bao XY (2018). Epidemiology of Moyamoya disease in China: Single-center population-based study. World Neurosurg..

[17] Guzman, R. *et al.* Clinical outcome after 450 revascularization procedures for moyamoya disease. *J. Neurosurg.* 927–935. 10.3171/2009.4.Jns081649 (2009). 10.3171/2009.4.JNS081649 19463046

[18] Kazumata K (2017). Direct anastomosis using occipital artery for additional revascularization in Moyamoya disease after combined superficial temporal artery-middle cerebral artery and indirect bypass. Oper. Neurosurg. (Hagerstown).

[19] Deng X (2018). Direct versus indirect bypasses for adult ischemic-type moyamoya disease: A propensity score-matched analysis. J. Neurosurg..

[20] Macyszyn L (2017). Direct versus indirect revascularization procedures for moyamoya disease: A comparative effectiveness study. J. Neurosurg..

[21] Park SE, Kim JS, Park EK, Shim KW, Kim DS (2018). Direct versus indirect revascularization in the treatment of moyamoya disease. J. Neurosurg..

[22] Kazumata K (2014). The frequency of postoperative stroke in moyamoya disease following combined revascularization: A single-university series and systematic review. J. Neurosurg..

[23] Teo MK, Madhugiri VS, Steinberg GK (2017). Editorial: Direct versus indirect bypass for moyamoya disease: ongoing controversy. J. Neurosurg..

[24] Guzman R (2009). Clinical outcome after 450 revascularization procedures for moyamoya disease: Clinical article. J. Neurosurg..

[25] Tashiro R (2019). Incidence and risk factors of the watershed shift phenomenon after superficial temporal artery-middle cerebral artery anastomosis for adult Moyamoya disease. Cerebrovasc. Dis..

[26] Hayashi T, Shirane R, Fujimura M, Tominaga T (2010). Postoperative neurological deterioration in pediatric moyamoya disease: Watershed shift and hyperperfusion. J. Neurosurg. Pediatr..

[27] Kim JE (2008). Transient hyperperfusion after superficial temporal artery/middle cerebral artery bypass surgery as a possible cause of postoperative transient neurological deterioration. Cerebrovasc. Dis..

[28] Kim T, Oh CW, Bang JS, Kim JE, Cho WS (2016). Moyamoya disease: Treatment and outcomes. J. Stroke.

[29] Rashad S, Fujimura M, Niizuma K, Endo H, Tominaga T (2016). Long-term follow-up of pediatric moyamoya disease treated by combined direct-indirect revascularization surgery: Single institute experience with surgical and perioperative management. Neurosurg. Rev..

[30] Wei W, Chen X, Yu J, Li XQ (2019). Risk factors for postoperative stroke in adults patients with moyamoya disease: A systematic review with meta-analysis. BMC Neurol..

[31] Hyun SJ, Kim JS, Hong SC (2010). Prognostic factors associated with perioperative ischemic complications in adult-onset moyamoya disease. Acta Neurochir. (Wien).

[32] Kim, S. K. *et al.* Moyamoya disease among young patients: Its aggressive clinical course and the role of active surgical treatment. *Neurosurgery* **54**, 840–844 (discussion 844–846). 10.1227/01.neu.0000114140.41509.14 (2004). 10.1227/01.neu.0000114140.41509.14 15046649

[33] Czabanka M (2011). Proposal for a new grading of Moyamoya disease in adult patients. Cerebrovasc. Dis..

[34] Teo M (2020). Validation and application for the Berlin grading system of Moyamoya Disease in adult patients. Neurosurgery.

[35] Antonucci MU (2016). Acute preoperative infarcts and poor cerebrovascular reserve are independent risk factors for severe ischemic complications following direct extracranial-intracranial bypass for Moyamoya disease. AJNR Am. J. Neuroradiol..

[36] Onozuka, D. *et al.* Prehospital antiplatelet use and functional status on admission of patients with non-haemorrhagic moyamoya disease: A nationwide retrospective cohort study (J-ASPECT study). *BMJ Open* **6**, e009942. 10.1136/bmjopen-2015-009942 (2016). 10.1136/bmjopen-2015-009942 PMC4800148 27008684

[37] Kanamori F (2021). Effects of aspirin and heparin treatment on perioperative outcomes in patients with Moyamoya disease. Acta Neurochir. (Wien).

[38] Iwama, T., Hashimoto, N. & Yonekawa, Y. The relevance of hemodynamic factors to perioperative ischemic complications in childhood moyamoya disease. *Neurosurgery* **38**, 1120–1125 (discussion 1125–1126) (1996). 10.1097/00006123-199606000-00011 8727141

[39] Sato K, Shirane R, Yoshimoto T (1997). Perioperative factors related to the development of ischemic complications in patients with moyamoya disease. Child's Nerv. Syst. ChNS.

[40] Soriano SG, Sethna NF, Scott RM (1993). Anesthetic management of children with moyamoya syndrome. Anesth. Analg..

[41] Eisenberg HM, Frankowski RF, Contant CF, Marshall LF, Walker MD (1988). High-dose barbiturate control of elevated intracranial pressure in patients with severe head injury. J. Neurosurg..

[42] Smith AL, Marque JJ (1976). Anesthetics and cerebral edema. Anesthesiology.

[43] Smith AL, Hoff JT, Nielsen SL, Larson CP (1974). Barbiturate protection in acute focal cerebral ischemia. Stroke.

[44] Zhai X (2018). Risk factors associated with neurologic deterioration after combined direct and indirect revascularization in patients with Moyamoya disease on the East Coast of China. World Neurosurg..

