# Supplementary material for: Risk factors and a novel cerebral infarction extent scoring system for postoperative cerebral ischemia in patients with ischemic Moyamoya disease
Source: Sci Rep. 2023 Apr 7;13:5726. doi: 10.1038/s41598-022-26985-3 (PMC10082086; doi:10.1038/s41598-022-26985-3)
Supplement: Supplementary file 1 — Supplementary Information. [file 41598_2022_26985_MOESM1_ESM.pdf]

Supplementary Table 1. Clinical characteristics of pediatric MMD patients

Characteristic	All Pts(n=36)	Post-op Ischemic Complications		*p* Value
		Absent (n =30)	Present (n =6)	
Gender				
Male	22	19	3	0.658
Female	14	11	3	
Onset symptoms				
TIA	17	16	1	0.650
Infarction	19	28	5	
PCA Involvement	12	9	3	0.378
Suzuki Stage				
1	0	0	0	0.910
2	6	5	1	
3	18	15	3	
4	10	15	2	
5	2	2	0	
6	0	0	0	
CIES	2.8±0.6	6.7±1.7	2.1±0.5	0.002
Interval btw infarction and op				
<8w	11	6	5	0.006
>=8w	25	24	1	
Type of surgery				
Indirect bypass	36	30	6	
Operating duration	110.1±6.9	104.7±4.6	136.7±35.2	0.086
Strict Peri-op management	28	26	2	0.014
mRS at 3 mths Post-op	1.9±1.3	1.4±0.2	3.7±0.6	<0.0001

CIES: Cerebral Infarction Extent Scoring System; Interval btw infarction and op: Interval time between frequent TIA or infarction presentation and operation. mRS: modified Rankin Scale; TIA: Transient ischemic attack; PCA: Posterior cerebral artery; Peri-op: Perioperative; Post-op: Postoperative.

Supplementary Table 2. Clinical characteristics of adult MMD patients

Characteristic	All Pts(n=34)	Post-op Ischemic Complications		*p* Value
		Absent (n =25)	Present (n =9)	
Gender				
Male	22	18	4	0.224
Female	12	7	5	
Onset symptoms				
TIA	6	6	0	0.162
Infarction	28	19	9	
PCA Involvement	10	5	5	0.085
Suzuki Stage				
1	0	0	0	0.185
2	7	4	3	
3	14	12	2	
4	12	9	3	
5	1	0	1	
6	0	0	0	
CIES	3.8±0.5	2.8±0.4	6.3±1.4	0.001
Interval btw infarction and op				
<8w	8	5	3	0.649
>=8w	26	20	6	
Type of surgery				
Indirect bypass	14	9	5	0.435
Combined bypass	20	16	4	
Operating duration	225.4±18.1	222.9±18.8	232.2±46.5	0.824
Strict Peri-op management	25	21	4	0.021
mRS at 3 mths Post-op	2.1±0.2	1.6±0.2	3.2±0.5	0.0005

CIES: Cerebral Infarction Extent Scoring System; Interval btw infarction and op: Interval time between frequent TIA or infarction presentation and operation. mRS: modified Rankin Scale; TIA: Transient ischemic attack; PCA: Posterior cerebral artery; Peri-op: Perioperative; Post-op: Postoperative.
